# Role of fibroblasts in nonfibrotic autoimmune skin diseases

**DOI:** 10.1186/s10020-024-00949-x

**Published:** 2024-10-17

**Authors:** Yuexi He, Zhenxin Han, Qiuli Zhang, Lin Liu, Jianmin Chang

**Affiliations:** 1 Department of Dermatology, Sichuan Provincial People’s Hospital, University of Electronic Science and Technology of China, Chengdu, 610000 China; 2 Department of Dermatology, Beijing Hospital, National Center of Gerontology, Institute of Geriatric Medicine, Chinese Academy of Medical Sciences & Peking Union Medical College, Beijing, 100730 China; 3 Medical School, University of Chinese Academy of Sciences, Beijing, 101408 China

**Keywords:** Fibroblasts, Autoimmune skin diseases, Psoriasis, Vitiligo, Atopic dermatitis

## Abstract

Autoimmune diseases, a disease characterized by immune imbalance caused by the human immune system mistakenly attacking its own components, include vitiligo, psoriasis and atopic dermatitis (AD). Previous studies on autoimmune diseases have focused mainly on immune cells, keratinocytes and endothelial cells. Fibroblasts, the main cells that secrete the extracellular matrix (ECM) in the dermis, have been studied thoroughly in terms of fibrosis and wound healing. However, an increasing number of studies have shown that fibroblasts play an important role in nonfibrotic autoimmune skin diseases. In this article, the previously reported role of fibroblasts in nonfibrous autoimmune skin diseases such as psoriasis, vitiligo and AD is summarized to provide new ideas for the treatment of this disease.

## Introduction

Autoimmune disease is a disease caused by primary immune tolerance and an altered immune response to autoantigens. It is characterized by an immune imbalance because the human immune system mistakenly attacks its own components and causes psoriasis, vitiligo, atopic dermatitis (AD), systemic sclerosis, sclerosing moss and a series of diseases. Lymphocytes (Seiringer et al. 2022; Bruyn Carlier et al. 2021), keratinocytes (Zhou et al. 2022; Li et al. 2017) and endothelial cells (Li et al. 2023) are commonly studied in the context of autoimmune skin diseases.

Dermal fibroblasts support skin functions and promote wound healing by secreting and remodeling the extracellular matrix (ECM). An increasing number of studies have focused on its role in fibrotic diseases, such as keloids and systemic sclerosis. However, with increasingresearch and the popularization of single-cell sequencing technology, an increasing number of researchers have begun to investigate the role of fibroblasts in autoimmune skin diseases. In this review, we summarize the mechanisms of fibroblasts in nonfibrotic autoimmune skin diseases, such as psoriasis, vitiligo and AD.

## Psoriasis

Psoriasis is a common erythematous and scaly skin disease with a genetic background that is related to an abnormal immune response.

### Changes at the transcriptome level

Griva (Grivas et al. 2022) used a single-cell transcriptome technique to show that fibroblasts proliferate actively in the lesions of psoriatic patients with arthropathy and interact with immune cells in the peripheral blood through signaling pathways such a the WNT, NOTCH, and PDGF pathways. Through single-cell transcriptomic and spatial transcriptomic techniques, Ma (Ma et al. 2023) further revealed that in psoriasis, SFRP2^+^ fibroblasts transition from a fibrotic state to an inflammatory state by producing C-C motif chemokine ligand (CCL) 13, CCL19, C-X-C motif chemokine ligand (CXCL) 1, and CXCL12. The following spatially similar cell types were affected: CCR2^+^ myeloid cells, CCR7^+^ LAMP3^+^ dendritic cells, neutrophils, CXCR4^+^ CD8^+^ Tc17 cells and CXCR4^+^ keratinocytes. Furthermore, promoting interleukin (IL)-17 production enhances the inflammatory response to IL-17A and tumor necrosis factor (TNF) in the epidermis. Fibroblasts from psoriatic lesions maintained their unique transcriptomic characteristics, including endothelial cell activation, histone exchange and chromatin silencing, within 4 generations after isolation. These findings suggest that fibroblasts can maintain their transcriptional abnormalities through epigenetic modifications (Kim et al. 2023).

### Changes in protein levels

The upregulation of the expression of proinflammatory factors [S100 calcium binding protein (S100)A8/9, nuclear factor kappa-B (NF-κb), and TNF-α] and antisenescent proteins, as well as signal transduction- and proteolysis-related proteins, by fibroblasts was detected via proteomics in psoriatic lesions. The downregulated proteins are responsible for biological processes such as transcription/translation and glycolysis/ATP synthesis. S100A8/A9 activates keratinocyte complement component protein 3 (C3) in psoriasis. After activation, C3 stimulates the production of cytokines, interleukins and growth factors by immune cells (Schonthaler et al. 2013). Previous studies have suggested that the S100A protein in psoriatic tissues is synthesized by activated macrophages under inflammatory conditions (Batycka-Baran et al. 2015), and the role of fibroblasts in S100A8/A9 synthesis in psoriasis is poorly understood; however, the levels of these proteins are significantly increased in dermal fibroblasts, suggesting a specific role for fibroblasts in proinflammatory signaling leading to hyperproliferation of keratinocytes in psoriasis. NF-κB not only induces the expression of inflammatory factors but also stimulates the secretion of other proinflammatory factors such as TNF-α. TNF-α promotes keratinocyte proliferation and macrophage migration and enhances the signaling of inflammatory cytokines between cells (Marble et al. 2007; Kristensen et al. 1993). In patients with psoriasis, oxidative stress is involved in the disease process. The increased expression of antiaging proteins in fibroblasts suggests that they may limit the body’s oxidative response. These changes in fibroblasts may directly affect intercellular signaling and promote the excessive proliferation of epidermal keratinocytes (Gęgotek et al. 2020).

Sidler (Sidler et al. 2017) reported that fibroblasts can respond to the effects of tumor necrosis factor-like weak inducer of apoptosis (TWEAK) by expressing IL-19, which induces the expression of thymic stromal lymphopoietin (TSLP) and regulates many chemokines to affect the development of psoriasis and AD.

### Cell-cell interactions

Ceramide plays a crucial role in the formation and maintenance of the integrity of the skin barrier, with 50% being lipid types. Ceramide and its metabolites are also involved in cell signaling and are involved in the proliferation, differentiation and apoptosis of human epidermal cells (Coderch et al. 2003; Candi et al. 2005). In psoriasis, a reduction in ceramide synthesis and epidermal levels has previously been found to be positively associated with the Psoriasis Area and Severity Index (PASI) score in patients with mild to moderate psoriasis (Lew et al. 2006). There was no significant difference in ceramide content between keratinocytes and fibroblasts in the disease group and those in the healthy control group. However, there was a significant difference in the classes of ceramides, and the levels of esterified ω-hydroxy fatty acid sphingosine were significantly increased in the fibroblasts of the disease group (Łuczaj et al. 2020). The tendency for most ceramide observed in fibroblasts to be upregulated may be that the cells respond to changes in the barrier during skin inflammation. The increased production of ceramide leads to the induction of exosome secretion (Lew et al. 2006). The differences in ceramide levels between keratinocytess and fibroblasts may be related to the different signaling functions of ceramide species in the dermis and epidermis. Esterified ω-hydroxy fatty acid sphingosine is important for epidermal barrier function (Uchida and Holleran 2008) and is the most distinct type of fibroblast that can distinguish between psoriatic patients and healthy subjects.

Syndecan-1 is involved in the development of inflammatory diseases (Gopal 2020). In psoriasis, fibroblasts promote keratinocyte expression of Syndecan-1, which is highly expressed at the basal cell membrane and intercellular junctions (Koliakou et al. 2022).

## Vitiligo

Vitiligo is a common autoimmune skin disease. The typical clinical manifestation of vitiligo is a milky white patch that can appear on the edge of any part of the body, which affects the patient’s physical and mental health.

### Autophagy and aging

The upregulation of autophagosome- and autophagy-related gene expression, which is characteristic of accelerated autophagy, was found in fibroblasts from nonlesional regions of patients with stable vitiligo. Autophagy can reduce the risk of cell senescence, and the expression of senescence markers is upregulated after autophagy is inhibited in fibroblasts. Autophagy, which occurs in fibroblasts, may be an adaptive response to persistent metabolic changes that contribute to the recovery of intracellular glucose, amino acid, and lipid levels and antagonize the progression of vitiligo (Bastonini et al. 2021). Another study revealed that in patients with nonsegmental vitiligo, the number of β-galactosidase-positive fibroblasts in the lesions was significantly greater than that in the nonlesional and control groups, and the expression of the senescence markers p16, p21 and hp1 was greater in the fibroblasts of skin lesions (Rani et al. 2017). β-Galactosidase is a marker of aging and is independent of the DNA synthesis process (Collado et al. 2005). Stress-induced senescence is mediated mainly by the activation of P16 (Dickson et al. 2000). P21 also inhibits cell cycle progression by downregulating kinases, known as cyclin-dependent kinases (Harper et al. 1995). Stress-induced senescent fibroblasts may reduce the secretion of important growth factors such as BFGF and neuromodulin, which are essential for melanocyte viability and function. This leads to the loss of melanocytes and the occurrence of pigmented vitiligo.

Many cells with a myofibroblast phenotype are found in the dermis of vitiligo patients. The appearance of myofibroblasts may be a consequence of dysregulated interactions between mediators and messengers released throughout the skin, and elevated levels of basal reactive oxygen species may be a key factor. Mitochondria are thought to be the site of increased reactive oxygen species production in vitiligo, whose functional impairment leads to high basal intracellular reactive oxygen species production, and may also serve as an intrinsic defect in dermal fibroblasts; in turn, these defects promote the differentiation of myofibroblasts. Fibroblasts carrying dysfunctional mitochondrial complex I were found in previous studies to produce high levels of reactive oxygen species and to correlate with elevated α-SMA expression, thereby promoting their transformation into myofibroblasts (Taddei et al. 2012).

The levels of cholesterol and oxysterols in vitiligo fibroblasts are significantly greater than those in normal fibroblasts (Kovacs et al. 2018). Oxysterols participate in the development of a myofibroblast phenotype by causing an unbalanced redox state in the dermis and promoting the induction of premature senescence, suggesting that stress induces premature senescence of fibroblasts.

### Recruitment of T cells

The disease is usually characterized by bilateral symmetry in certain anatomical areas of the body. On the basis of these findings, researchers have shown that the location preference of vitiligo is associated with the ability of fibroblasts at different sites to respond to interferon gamma (IFN-γ), and as the only skin cells capable of recruiting and activating their own active CD8^+^ T cells, the ability of these cells to recruit active CD8^+^ T cells via CXCL9/CXCL10-CXCR3 correlates with IFN-γ (Xu et al. 2022). Jin (Jin et al. 2023) reported that fibroblasts bound to IFN-γ promote the production of the chemokines CCL2 and CCL8, which in turn promote the differentiation of naive T cells into Th2 cells. CCL8 attracts Th2 cells through the JAK-ATK pathway, which can be blocked by JAK inhibitors. In the clinic, JAK inhibitors have also achieved good efficacy, suggesting that they may promote the recovery of vitiligo patients in a variety of ways.

### Affecting melanocytes

Fibroblasts can also affect the growth and migration of melanocytes via paracrine signaling. Dickkopf WNT signaling pathway inhibitor 1 (DKK1) is significantly more highly expressed in the hypochromic region than in the nonhypochromic region, and its expression is increased in palmoplantar fibroblasts (Yamaguchi et al. 2008). Increased expression of DKK1 in fibroblast cultures from nondiseased areas suggests dysregulation of the mediator throughout the skin (Kovacs et al. 2018). DKK1 was found to be involved in the formation of palmoplantar skin by inhibiting the function and growth of melanocytes and regulating the β-catenin/MITF pathway. DKK1 can also inhibit the production of protease activated receptor 2 (PAR2) in keratinocytes to reduce melanin transfer to keratinocytes (Chen et al. 2024; Oh et al. 2012) and inhibit E-cadherin expression in melanocytes, attenuating the adhesion of melanocytes to adjacent keratinocytes and thereby shedding (Kovacs et al. 2018). However, altered E-cadherin levels in melanocytes are also associated with increased numbers of bioactive messengers released by fibroblasts, in which both increased HGF and ET-1 downregulate E-cadherin in melanocytes (Kovacs et al. 2018; Haass and Herlyn 2005). Thus, fibroblasts may play an important role in melanocyte death in vitiligo.

## Atopic dermatitis

AD is a chronic, recurrent, and allergic disease related to inflammatory skin lesions with a genetic component.

### Immunity and inflammation

NF-κB activation is critical for regulating inflammatory immune responses, and inhibitor of nuclear factor kappa-B kinase subunit beta (Ikkb) is a gene required for activation of the canonical NF-κB pathway. When activation of the canonical NF-κB pathway in Prx1^+^ skin fibroblasts is blocked by deletion of the Ikkb gene, cutaneous manifestations of AD occur, leading simultaneously to an increase in CCL11 expression (Ko et al. 2022). The upregulation of CCL11 was associated with human AD skin (Owczarek et al. 2010), and dermal fibroblasts from human atopic skin presented increased CCL11 mRNA expression in response to IL-4 stimulation in vitro compared with that in controls (Gahr et al. 2011). CCL11 overexpression causes human atopic dermatitis-like skin lesions such as eosinophil granulocyte infiltration and a subsequent Th2 immune response (Weidinger et al. 2018).

The interaction between eosinophils and dermal fibroblasts is essential for triggering AD. In cocultures of eosinophils and dermal fibroblasts, IL-37b inhibited IL-31-and IL-33-induced increases in TNF-α, IL-6, CXCL8, CCL2, and CCL5 in vitro. The supernatants of the eosinophil and dermal fibroblast coculture system produced higher levels of inflammatory cytokines and chemokines than did those of fibroblasts cultured alone; these results suggest that the interaction between eosinophils and fibroblasts may play an important role in the exacerbation of the inflammatory cascade (Hou et al. 2020). In this process IL-37b enhances autophagy mechanisms by regulating the AMPK‒mTOR signaling pathway, which inhibits IL-31-and IL-33-mediated inflammatory responses (Levine et al. 2011).

The expression of chemokines (CCL8, CCL2, and CXCL5) and molecules involved in tissue remodeling (TNC, PPSTN, COL5A2, and COL6A3) increased in fibroblasts from ovalbumin-induced allergic mice. Elevated expression of both IL-33 and PRG4 can inhibit the activation of NF-κB (Iqbal et al. 2016; Ali et al. 2011), suggesting that fibroblasts may play a role in susceptibility to skin infection in patients with AD (Leyva-Castillo et al. 2022).

### Cell-cell interactions

The expression of CXCR4 and its homologous ligand CXCL12 is obviously upregulated in the skin of AD patients. CXCR4^+^ skin-resident NKT cells are pathogenic tissue-resident memory T cells (TRMs), and fibroblasts are the main cells that secrete CXCL12 in AD. CXCR4^+^ NKT cells patrol and attach to CXCL12 cells, forming CXCR4^+^ NKT/CXCL12^+^ cell clusters in AD skin and promoting the development of allergic inflammation in AD skin (Sun et al. 2021).

Three major fibroblast phenotypes were identified via single-cell transcriptome techniques in which lesional and nonlesional skin was compared with normal skin in AD patients. Among them, the COL6A5^+^ COL18A1^+^ fibroblast subsets expressing inflammatory factors (CCL2, CCL19, CCL26, and IL-32) are unique to AD patients and are concentrated mainly at the dermal-epidermal junction. COL6A5 is a susceptibility gene for AD that may lead to abnormal fibroblast adhesion, collagen synthesis and metabolism, and barrier disruption (Sabatelli et al. 2011). COL18A1 effectively inhibits angiogenesis and binds to several other ECM components, leading to ECM disorganization in AD (Suzuki et al. 2009).

Thereafter, highly expressed CCL19 and CCL2 in these fibroblasts interact with CCR7, CCR1, and CCR2 on T cells and macrophage-dendritic cells to promote T-cell trafficking, lymphoid tissue, and type 2 cell recruitment (He et al. 2020; Gu et al. 2000; Takamura et al. 2007).

AD fibroblasts can promote the proliferation of healthy keratinocytes in organoids, mainly by thickening the basal and cuticle layers and decreasing the expression of differentiation markers such as microfilaments. Normal fibroblasts can repair the undifferentiated and disorganized epidermal cells produced by AD keratinocytes and promote the ability of the epidermis to differentiate into the normal basal layer, spinous layer and cuticle. Increased expression of terminal differentiation markers such as filaggrin and loricrin and a lack of leukemia inhibitory factor (LIF) are important causes of the aberrant effects of AD fibroblasts on the epidermis (Berroth et al. 2013). Filaggrin is an important molecule in the formation of cornified envelopes. Mutations in the filaggrin gene are associated with AD. Fibroblasts secrete LIF through the JAK-STAT3 pathway, which induces a decrease in the number of keratinocytes and epidermal structural changes (McKenzie and Szepietowski 2004; Wu et al. 2003).

In another organoid study (Löwa et al. 2020) of AD fibroblasts and normal keratinocytes, TSLP and PAR2 expression was significantly upregulated in the epidermal proliferation group, LIF expression was downregulated, and CD4^+^ T cells were recruited into the dermis. T-cell migration may be directly regulated by the stimulation of high TSLP levels in the epidermal proliferation group. In addition to T-cell migration, the molecular levels of IL-13, IL-6 and TNF-α, which are involved in Th2-mediated immune responses, are also elevated. In contrast to the inflammatory pattern observed in psoriasis, inflammatory changes are most pronounced in fibroblasts, pericytes, and keratinocytes in AD, and fibroblasts interact with Th2/Th22 cells via CXCL12 (Zhang et al. 2023).

## Other diseases

In skin fibroblasts, the expression of various inflammatory factors, such as SERPINB2, TNFRSF18, IL-33, CCL20, IL1RL1, CXCL3/5/8, and ICAM1, is upregulated after stimulation with IL-11, and the upregulated expression of IL8, IL6, MCP1, CCL20 and CXCL156 by chemotactic neutrophils, monocytes and lymphocytes promotes the development of inflammation (Widjaja et al. 2022). Dermal fibroblasts can be divided into four types: secretory-papillary (APCDD1, AXIN2, COLEC12, PTGDS and COL18A1), secretory-reticular (SLPI, CTHRC1, MGP and MFAP5), proinflammatory (CXCL2, CXCL3, CCL19 and APOE), and mesenchymal (ASPN, POSTN and GPC3). Among them, inflammatory fibroblast subsets are associated with the negative regulation of inflammatory responses, cellular chemotaxis, and cell proliferation; thus, fibroblasts may play a role in inflammatory skin diseases (Solé-Boldo et al. 2020).

## Conclusion

An increasing number of studies have shown that fibroblasts play important roles not only in fibrotic diseases but also in nonfibrotic autoimmune skin diseases. In this article, we review the roles of fibroblasts in autoimmune skin diseases such as psoriasis, vitiligo and AD, including the secretion of inflammatory factors and chemotactic immune cells and interactions with keratinocytes, endothelial cells, and dendritic cells, which influence disease progression. Single-cell transcriptome technology is very useful for researchers to discover new cell subsets and explore their functions. With the innovation of space science and proteomics, more studies on fibroblasts will be presented, and the results will provide new ideas for exploring the mechanism of diseases and finding therapeutic targets.

## Data Availability

Not applicable.
